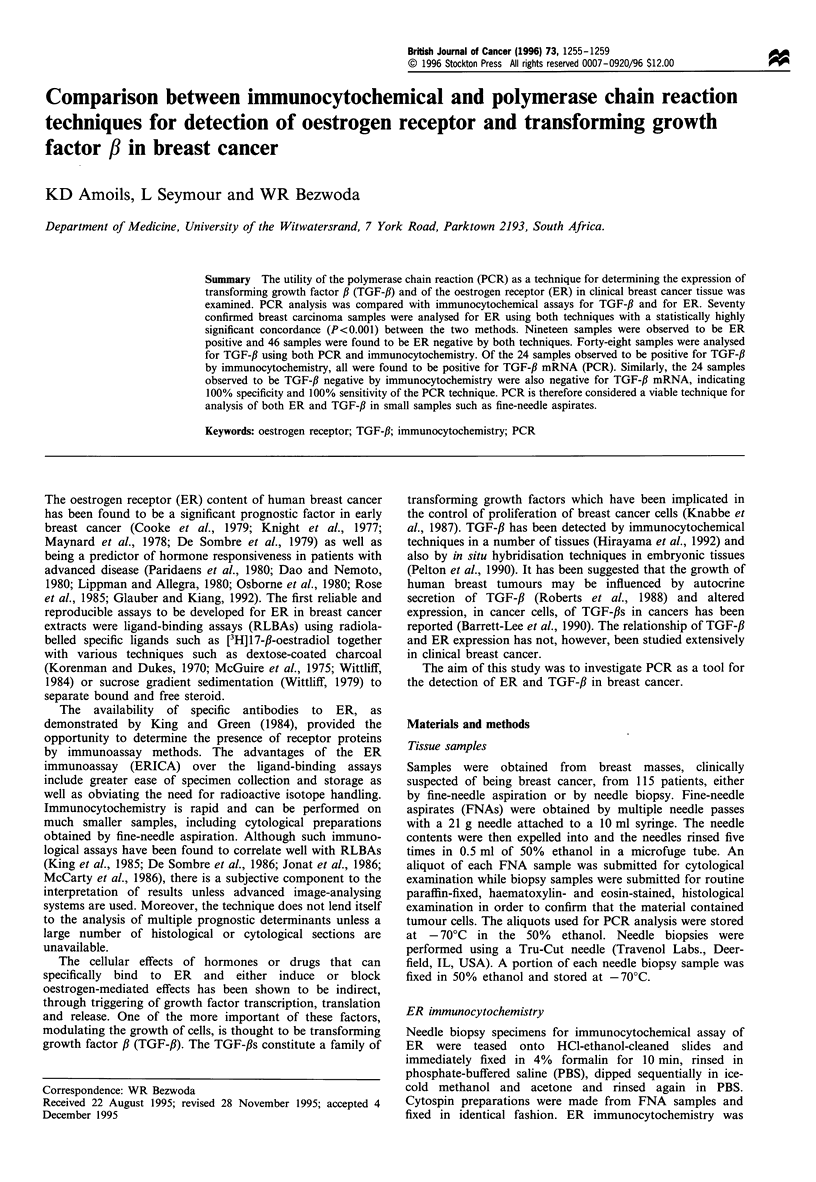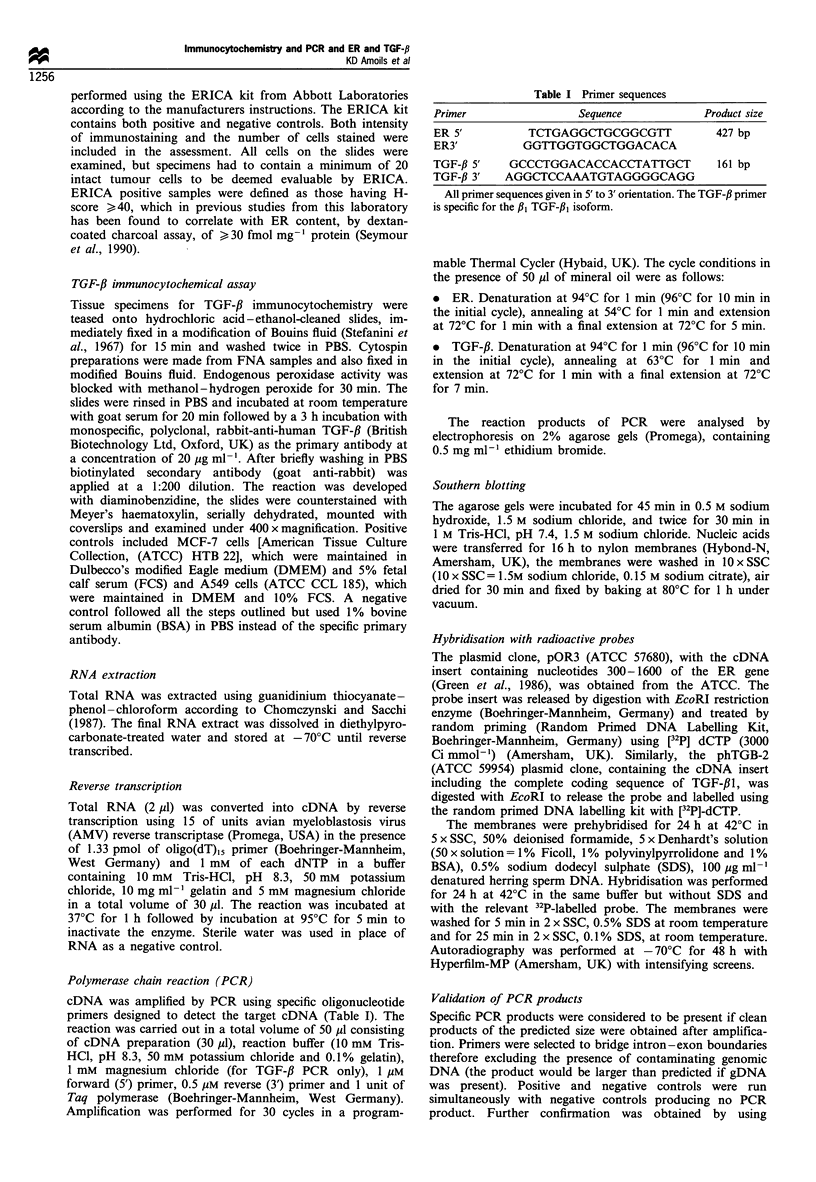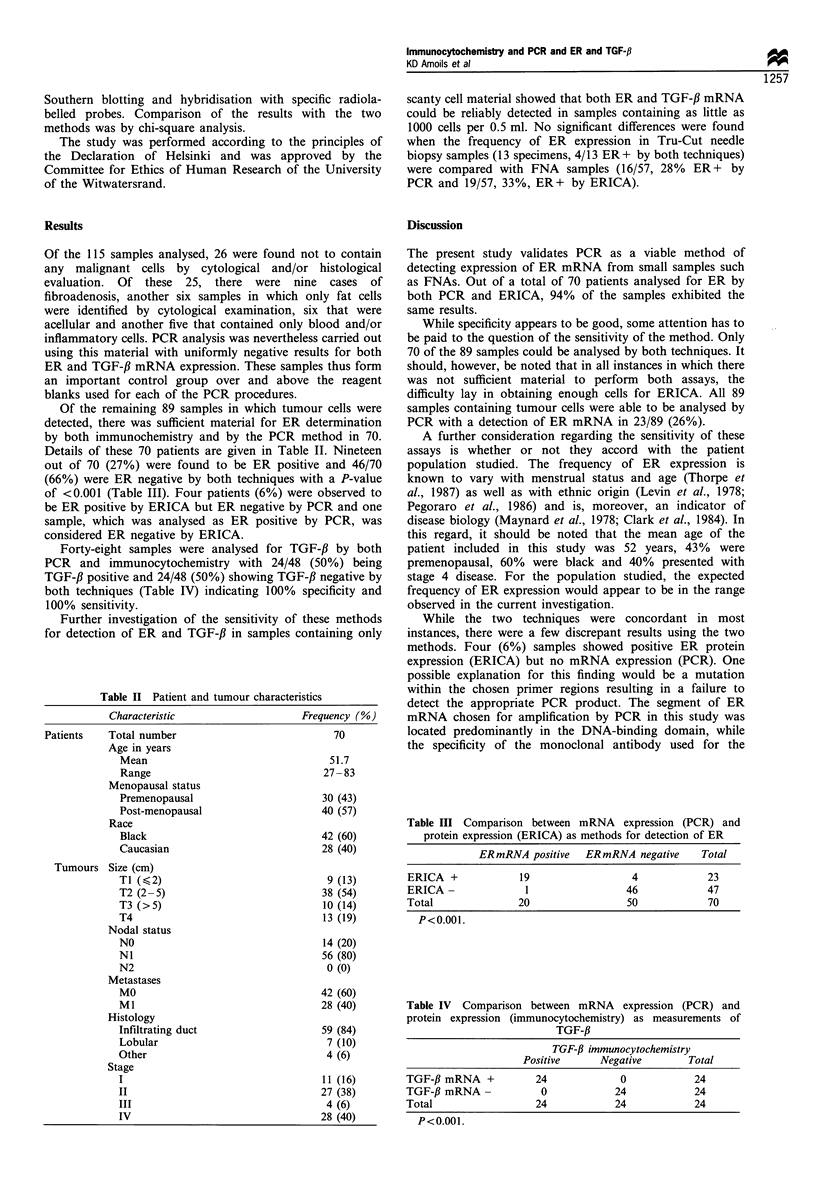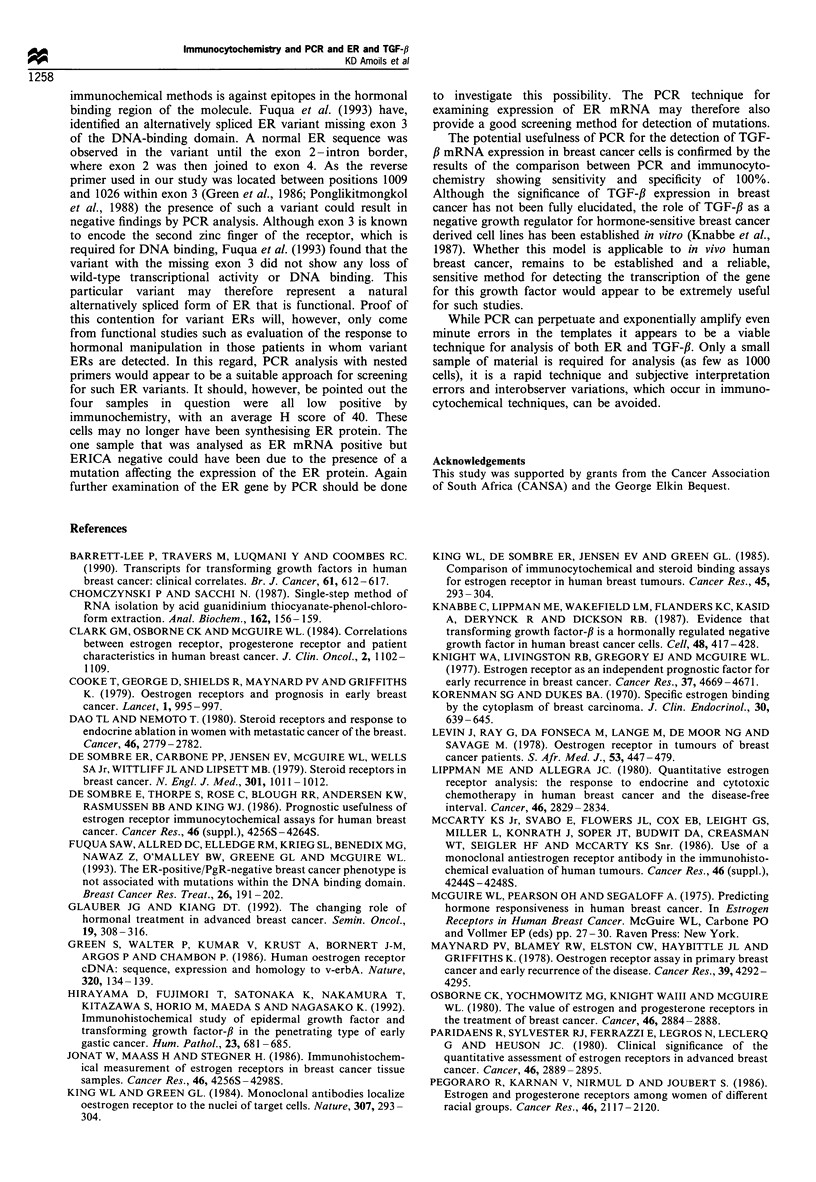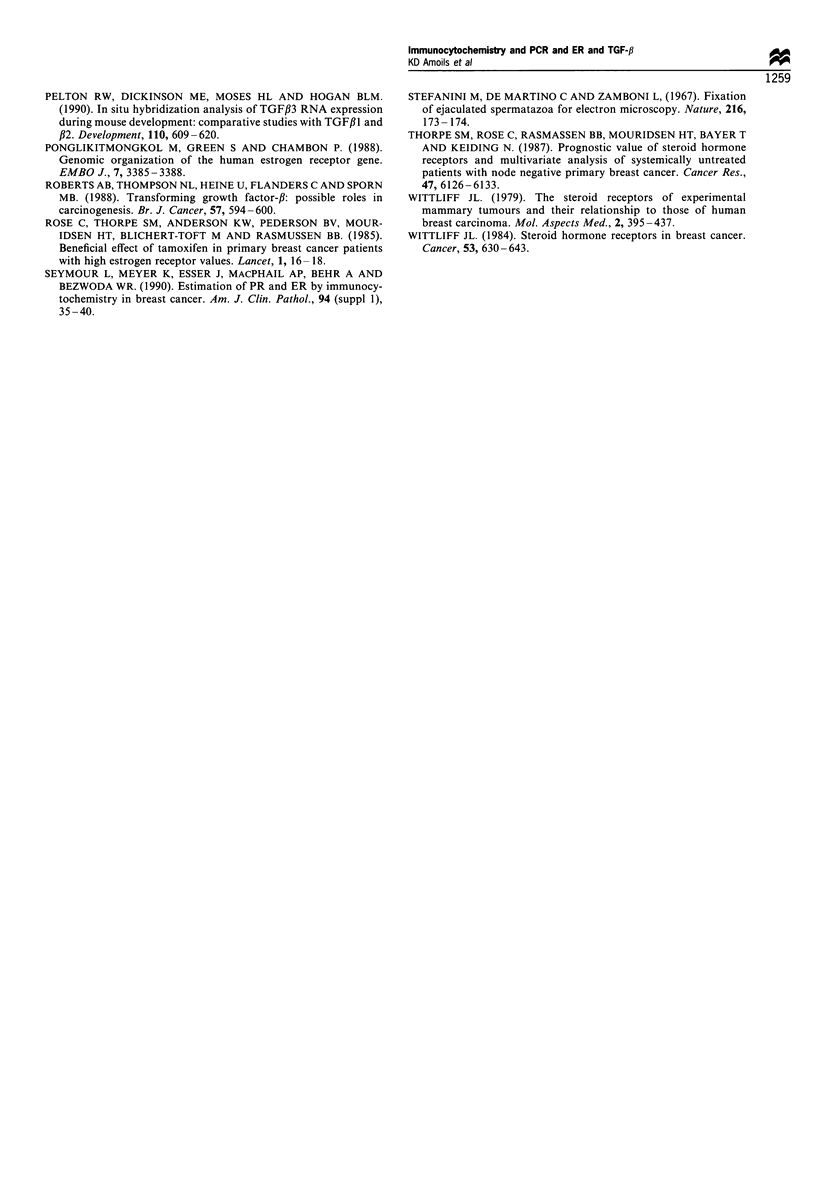# Comparison between immunocytochemical and polymerase chain reaction techniques for detection of oestrogen receptor and transforming growth factor beta in breast cancer.

**DOI:** 10.1038/bjc.1996.240

**Published:** 1996-05

**Authors:** K. D. Amoils, L. Seymour, W. R. Bezwoda

**Affiliations:** Department of Medicine, University of Witwatersrand, Parktown, South Africa.

## Abstract

The utility of the polymerase chain reaction (PCR) as a technique for determining the expression of transforming growth factor beta (TGF-beta) and of the oestrogen receptor (ER) in clinical breast cancer tissue was examined. PCR analysis was compared with immunocytochemical assays for TGF-beta and for ER. Seventy confirmed breast carcinoma samples were analysed for ER using both techniques with a statistically highly significant concordance (P < 0.001) between the two methods. Nineteen samples were observed to be ER positive and 46 samples were found to be ER negative by both techniques. Forty-eight samples were analysed for TGF-beta using both PCR and immunocytochemistry. Of the 24 samples observed to be positive for TGF-beta by immunocytochemistry, all were found to be positive for TGF-beta mRNA (PCR). Similarly, the 24 samples observed to be TGF-beta negative by immunocytochemistry were also negative for TGF-beta mRNA, indicating 100% specificity and 100% sensitivity of the PCR technique. PCR is therefore considered a viable technique for analysis of both ER and TGF-beta in small samples such as fine-needle aspirates.


British Journal of Cancer (1996) 73, 1255-1259
© 1996 Stockton Press All rights reserved 0007-0920/96 $12.00

### Comparison between immunocytochemical and polymerase chain reaction techniques for detection of oestrogen receptor and transforming growth factor β in breast cancer

KD Amoils, L Seymour and WR Bezwoda

*Department of Medicine, University of the Witwatersrand, 7 York Road, Parktown 2193, South Africa.*

**Summary** The utility of the polymerase chain reaction (PCR) as a technique for determining the expression of transforming growth factor β (TGF-β) and of the oestrogen receptor (ER) in clinical breast cancer tissue was examined. PCR analysis was compared with immunocytochemical assays for TGF-β and for ER. Seventy confirmed breast carcinoma samples were analysed for ER using both techniques with a statistically highly significant concordance ($P<0.001$) between the two methods. Nineteen samples were observed to be ER positive and 46 samples were found to be ER negative by both techniques. Forty-eight samples were analysed for TGF-β using both PCR and immunocytochemistry. Of the 24 samples observed to be positive for TGF-β by immunocytochemistry, all were found to be positive for TGF-β mRNA (PCR). Similarly, the 24 samples observed to be TGF-β negative by immunocytochemistry were also negative for TGF-β mRNA, indicating 100% specificity and 100% sensitivity of the PCR technique. PCR is therefore considered a viable technique for analysis of both ER and TGF-β in small samples such as fine-needle aspirates.

**Keywords:** oestrogen receptor; TGF-β; immunocytochemistry; PCR

The oestrogen receptor (ER) content of human breast cancer has been found to be a significant prognostic factor in early breast cancer (Cooke *et al.*, 1979; Knight *et al.*, 1977; Maynard *et al.*, 1978; De Sombre *et al.*, 1979) as well as being a predictor of hormone responsiveness in patients with advanced disease (Paridaens *et al.*, 1980; Dao and Nemoto, 1980; Lippman and Allegra, 1980; Osborne *et al.*, 1980; Rose *et al.*, 1985; Glauber and Kiang, 1992). The first reliable and reproducible assays to be developed for ER in breast cancer extracts were ligand-binding assays (RLBAs) using radiolabelled specific ligands such as $[^3H]17$-β-oestradiol together with various techniques such as dextose-coated charcoal (Korenman and Dukes, 1970; McGuire *et al.*, 1975; Wittliff, 1984) or sucrose gradient sedimentation (Wittliff, 1979) to separate bound and free steroid.

The availability of specific antibodies to ER, as demonstrated by King and Green (1984), provided the opportunity to determine the presence of receptor proteins by immunoassay methods. The advantages of the ER immunoassay (ERICA) over the ligand-binding assays include greater ease of specimen collection and storage as well as obviating the need for radioactive isotope handling. Immunocytochemistry is rapid and can be performed on much smaller samples, including cytological preparations obtained by fine-needle aspiration. Although such immunological assays have been found to correlate well with RLBAs (King *et al.*, 1985; De Sombre *et al.*, 1986; Jonat *et al.*, 1986; McCarty *et al.*, 1986), there is a subjective component to the interpretation of results unless advanced image-analysing systems are used. Moreover, the technique does not lend itself to the analysis of multiple prognostic determinants unless a large number of histological or cytological sections are unavailable.

The cellular effects of hormones or drugs that can specifically bind to ER and either induce or block oestrogen-mediated effects has been shown to be indirect, through triggering of growth factor transcription, translation and release. One of the more important of these factors, modulating the growth of cells, is thought to be transforming growth factor β (TGF-β). The TGF-βs constitute a family of transforming growth factors which have been implicated in the control of proliferation of breast cancer cells (Knabbe *et al.*, 1987). TGF-β has been detected by immunocytochemical techniques in a number of tissues (Hirayama *et al.*, 1992) and also by *in situ* hybridisation techniques in embryonic tissues (Pelton *et al.*, 1990). It has been suggested that the growth of human breast tumours may be influenced by autocrine secretion of TGF-β (Roberts *et al.*, 1988) and altered expression, in cancer cells, of TGF-βs in cancers has been reported (Barrett-Lee *et al.*, 1990). The relationship of TGF-β and ER expression has not, however, been studied extensively in clinical breast cancer.

The aim of this study was to investigate PCR as a tool for the detection of ER and TGF-β in breast cancer.

### Materials and methods

### *Tissue samples*

Samples were obtained from breast masses, clinically suspected of being breast cancer, from 115 patients, either by fine-needle aspiration or by needle biopsy. Fine-needle aspirates (FNAs) were obtained by multiple needle passes with a 21 g needle attached to a 10 ml syringe. The needle contents were then expelled into and the needles rinsed five times in 0.5 ml of 50% ethanol in a microfuge tube. An aliquot of each FNA sample was submitted for cytological examination while biopsy samples were submitted for routine paraffin-fixed, haematoxylin- and eosin-stained, histological examination in order to confirm that the material contained tumour cells. The aliquots used for PCR analysis were stored at −70°C in the 50% ethanol. Needle biopsies were performed using a Tru-Cut needle (Travenol Labs., Deerfield, IL, USA). A portion of each needle biopsy sample was fixed in 50% ethanol and stored at −70°C.

### *ER immunocytochemistry*

Needle biopsy specimens for immunocytochemical assay of ER were teased onto HCl-ethanol-cleaned slides and immediately fixed in 4% formalin for 10 min, rinsed in phosphate-buffered saline (PBS), dipped sequentially in ice-cold methanol and acetone and rinsed again in PBS. Cytospin preparations were made from FNA samples and fixed in identical fashion. ER immunocytochemistry was

Correspondence: WR Bezwoda
Received 22 August 1995; revised 28 November 1995; accepted 4 December 1995

performed using the ERICA kit from Abbott Laboratories according to the manufacturers instructions. The ERICA kit contains both positive and negative controls. Both intensity of immunostaining and the number of cells stained were included in the assessment. All cells on the slides were examined, but specimens had to contain a minimum of 20 intact tumour cells to be deemed evaluable by ERICA. ERICA positive samples were defined as those having H-score ⩾40, which in previous studies from this laboratory has been found to correlate with ER content, by dextan-coated charcoal assay, of ⩾30 fmol $mg^{-1}$ protein (Seymour *et al.*, 1990).

*TGF-β immunocytochemical assay*

Tissue specimens for TGF-β immunocytochemistry were teased onto hydrochloric acid–ethanol-cleaned slides, immediately fixed in a modification of Bouins fluid (Stefanini *et al.*, 1967) for 15 min and washed twice in PBS. Cytospin preparations were made from FNA samples and also fixed in modified Bouins fluid. Endogenous peroxidase activity was blocked with methanol–hydrogen peroxide for 30 min. The slides were rinsed in PBS and incubated at room temperature with goat serum for 20 min followed by a 3 h incubation with monospecific, polyclonal, rabbit-anti-human TGF-β (British Biotechnology Ltd, Oxford, UK) as the primary antibody at a concentration of 20 μg $ml^{-1}$. After briefly washing in PBS biotinylated secondary antibody (goat anti-rabbit) was applied at a 1:200 dilution. The reaction was developed with diaminobenzidine, the slides were counterstained with Meyer's haematoxylin, serially dehydrated, mounted with coverslips and examined under 400 × magnification. Positive controls included MCF-7 cells [American Tissue Culture Collection, (ATCC) HTB 22], which were maintained in Dulbecco's modified Eagle medium (DMEM) and 5% fetal calf serum (FCS) and A549 cells (ATCC CCL 185), which were maintained in DMEM and 10% FCS. A negative control followed all the steps outlined but used 1% bovine serum albumin (BSA) in PBS instead of the specific primary antibody.

*RNA extraction*

Total RNA was extracted using guanidinium thiocyanate–phenol–chloroform according to Chomczynski and Sacchi (1987). The final RNA extract was dissolved in diethylpyrocarbonate-treated water and stored at −70°C until reverse transcribed.

*Reverse transcription*

Total RNA (2 μl) was converted into cDNA by reverse transcription using 15 of units avian myeloblastosis virus (AMV) reverse transcriptase (Promega, USA) in the presence of 1.33 pmol of oligo$(dT)_{15}$ primer (Boehringer-Mannheim, West Germany) and 1 mM of each dNTP in a buffer containing 10 mM Tris-HCl, pH 8.3, 50 mM potassium chloride, 10 mg $ml^{-1}$ gelatin and 5 mM magnesium chloride in a total volume of 30 μl. The reaction was incubated at 37°C for 1 h followed by incubation at 95°C for 5 min to inactivate the enzyme. Sterile water was used in place of RNA as a negative control.

*Polymerase chain reaction (PCR)*

cDNA was amplified by PCR using specific oligonucleotide primers designed to detect the target cDNA (Table I). The reaction was carried out in a total volume of 50 μl consisting of cDNA preparation (30 μl), reaction buffer (10 mM Tris-HCl, pH 8.3, 50 mM potassium chloride and 0.1% gelatin), 1 mM magnesium chloride (for TGF-β PCR only), 1 μM forward (5′) primer, 0.5 μM reverse (3′) primer and 1 unit of *Taq* polymerase (Boehringer-Mannheim, West Germany). Amplification was performed for 30 cycles in a programmable Thermal Cycler (Hybaid, UK). The cycle conditions in the presence of 50 μl of mineral oil were as follows:

- ER. Denaturation at 94°C for 1 min (96°C for 10 min in the initial cycle), annealing at 54°C for 1 min and extension at 72°C for 1 min with a final extension at 72°C for 5 min.
- TGF-β. Denaturation at 94°C for 1 min (96°C for 10 min in the initial cycle), annealing at 63°C for 1 min and extension at 72°C for 1 min with a final extension at 72°C for 7 min.

**Table I** Primer sequences

| *Primer* | *Sequence* | *Product size* |
|---|---|---|
| ER 5′ | TCTGAGGCTGCGGCGTT | 427 bp |
| ER3′ | GGTTGGTGGCTGGACACA | |
| TGF-β 5′ | GCCCTGGACACCACCTATTGCT | 161 bp |
| TGF-β 3′ | AGGCTCCAAATGTAGGGGCAGG | |

All primer sequences given in 5′ to 3′ orientation. The TGF-β primer is specific for the $\beta_1$ TGF-$\beta_1$ isoform.

The reaction products of PCR were analysed by electrophoresis on 2% agarose gels (Promega), containing 0.5 mg $ml^{-1}$ ethidium bromide.

*Southern blotting*

The agarose gels were incubated for 45 min in 0.5 M sodium hydroxide, 1.5 M sodium chloride, and twice for 30 min in 1 M Tris-HCl, pH 7.4, 1.5 M sodium chloride. Nucleic acids were transferred for 16 h to nylon membranes (Hybond-N, Amersham, UK), the membranes were washed in 10 × SSC (10 × SSC = 1.5M sodium chloride, 0.15 M sodium citrate), air dried for 30 min and fixed by baking at 80°C for 1 h under vacuum.

*Hybridisation with radioactive probes*

The plasmid clone, pOR3 (ATCC 57680), with the cDNA insert containing nucleotides 300–1600 of the ER gene (Green *et al.*, 1986), was obtained from the ATCC. The probe insert was released by digestion with *Eco*RI restriction enzyme (Boehringer-Mannheim, Germany) and treated by random priming (Random Primed DNA Labelling Kit, Boehringer-Mannheim, Germany) using [$^{32}P$] dCTP (3000 Ci $mmol^{-1}$) (Amersham, UK). Similarly, the phTGB-2 (ATCC 59954) plasmid clone, containing the cDNA insert including the complete coding sequence of TGF-β1, was digested with *Eco*RI to release the probe and labelled using the random primed DNA labelling kit with [$^{32}P$]-dCTP.

The membranes were prehybridised for 24 h at 42°C in 5 × SSC, 50% deionised formamide, 5 × Denhardt's solution (50 × solution = 1% Ficoll, 1% polyvinylpyrrolidone and 1% BSA), 0.5% sodium dodecyl sulphate (SDS), 100 μg $ml^{-1}$ denatured herring sperm DNA. Hybridisation was performed for 24 h at 42°C in the same buffer but without SDS and with the relevant $^{32}P$-labelled probe. The membranes were washed for 5 min in 2 × SSC, 0.5% SDS at room temperature and for 25 min in 2 × SSC, 0.1% SDS, at room temperature. Autoradiography was performed at −70°C for 48 h with Hyperfilm-MP (Amersham, UK) with intensifying screens.

*Validation of PCR products*

Specific PCR products were considered to be present if clean products of the predicted size were obtained after amplification. Primers were selected to bridge intron–exon boundaries therefore excluding the presence of contaminating genomic DNA (the product would be larger than predicted if gDNA was present). Positive and negative controls were run simultaneously with negative controls producing no PCR product. Further confirmation was obtained by using

Southern blotting and hybridisation with specific radiolabelled probes. Comparison of the results with the two methods was by chi-square analysis.

The study was performed according to the principles of the Declaration of Helsinki and was approved by the Committee for Ethics of Human Research of the University of the Witwatersrand.

### Results

Of the 115 samples analysed, 26 were found not to contain any malignant cells by cytological and/or histological evaluation. Of these 25, there were nine cases of fibroadenosis, another six samples in which only fat cells were identified by cytological examination, six that were acellular and another five that contained only blood and/or inflammatory cells. PCR analysis was nevertheless carried out using this material with uniformly negative results for both ER and TGF-β mRNA expression. These samples thus form an important control group over and above the reagent blanks used for each of the PCR procedures.

Of the remaining 89 samples in which tumour cells were detected, there was sufficient material for ER determination by both immunochemistry and by the PCR method in 70. Details of these 70 patients are given in Table II. Nineteen out of 70 (27%) were found to be ER positive and 46/70 (66%) were ER negative by both techniques with a $P$-value of <0.001 (Table III). Four patients (6%) were observed to be ER positive by ERICA but ER negative by PCR and one sample, which was analysed as ER positive by PCR, was considered ER negative by ERICA.

Forty-eight samples were analysed for TGF-β by both PCR and immunocytochemistry with 24/48 (50%) being TGF-β positive and 24/48 (50%) showing TGF-β negative by both techniques (Table IV) indicating 100% specificity and 100% sensitivity.

Further investigation of the sensitivity of these methods for detection of ER and TGF-β in samples containing only scanty cell material showed that both ER and TGF-β mRNA could be reliably detected in samples containing as little as 1000 cells per 0.5 ml. No significant differences were found when the frequency of ER expression in Tru-Cut needle biopsy samples (13 specimens, 4/13 ER+ by both techniques) were compared with FNA samples (16/57, 28% ER+ by PCR and 19/57, 33%, ER+ by ERICA).

### Discussion

The present study validates PCR as a viable method of detecting expression of ER mRNA from small samples such as FNAs. Out of a total of 70 patients analysed for ER by both PCR and ERICA, 94% of the samples exhibited the same results.

While specificity appears to be good, some attention has to be paid to the question of the sensitivity of the method. Only 70 of the 89 samples could be analysed by both techniques. It should, however, be noted that in all instances in which there was not sufficient material to perform both assays, the difficulty lay in obtaining enough cells for ERICA. All 89 samples containing tumour cells were able to be analysed by PCR with a detection of ER mRNA in 23/89 (26%).

A further consideration regarding the sensitivity of these assays is whether or not they accord with the patient population studied. The frequency of ER expression is known to vary with menstrual status and age (Thorpe *et al.*, 1987) as well as with ethnic origin (Levin *et al.*, 1978; Pegoraro *et al.*, 1986) and is, moreover, an indicator of disease biology (Maynard *et al.*, 1978; Clark *et al.*, 1984). In this regard, it should be noted that the mean age of the patient included in this study was 52 years, 43% were premenopausal, 60% were black and 40% presented with stage 4 disease. For the population studied, the expected frequency of ER expression would appear to be in the range observed in the current investigation.

While the two techniques were concordant in most instances, there were a few discrepant results using the two methods. Four (6%) samples showed positive ER protein expression (ERICA) but no mRNA expression (PCR). One possible explanation for this finding would be a mutation within the chosen primer regions resulting in a failure to detect the appropriate PCR product. The segment of ER mRNA chosen for amplification by PCR in this study was located predominantly in the DNA-binding domain, while the specificity of the monoclonal antibody used for the

**Table II** Patient and tumour characteristics

| | *Characteristic* | *Frequency (%)* |
|---|---|---|
| Patients | Total number | 70 |
| | Age in years | |
| | Mean | 51.7 |
| | Range | 27–83 |
| | Menopausal status | |
| | Premenopausal | 30 (43) |
| | Post-menopausal | 40 (57) |
| | Race | |
| | Black | 42 (60) |
| | Caucasian | 28 (40) |
| Tumours | Size (cm) | |
| | T1 (⩽2) | 9 (13) |
| | T2 (2–5) | 38 (54) |
| | T3 (>5) | 10 (14) |
| | T4 | 13 (19) |
| | Nodal status | |
| | N0 | 14 (20) |
| | N1 | 56 (80) |
| | N2 | 0 (0) |
| | Metastases | |
| | M0 | 42 (60) |
| | M1 | 28 (40) |
| | Histology | |
| | Infiltrating duct | 59 (84) |
| | Lobular | 7 (10) |
| | Other | 4 (6) |
| | Stage | |
| | I | 11 (16) |
| | II | 27 (38) |
| | III | 4 (6) |
| | IV | 28 (40) |

**Table III** Comparison between mRNA expression (PCR) and protein expression (ERICA) as methods for detection of ER

| | *ERmRNA positive* | *ERmRNA negative* | *Total* |
|---|---|---|---|
| ERICA + | 19 | 4 | 23 |
| ERICA − | 1 | 46 | 47 |
| Total | 20 | 50 | 70 |

$P$<0.001.

**Table IV** Comparison between mRNA expression (PCR) and protein expression (immunocytochemistry) as measurements of TGF-β

| | *TGF-β immunocytochemistry* | | |
|---|---|---|---|
| | *Positive* | *Negative* | *Total* |
| TGF-β mRNA + | 24 | 0 | 24 |
| TGF-β mRNA − | 0 | 24 | 24 |
| Total | 24 | 24 | 24 |

$P$<0.001.

immunochemical methods is against epitopes in the hormonal binding region of the molecule. Fuqua *et al.* (1993) have, identified an alternatively spliced ER variant missing exon 3 of the DNA-binding domain. A normal ER sequence was observed in the variant until the exon 2–intron border, where exon 2 was then joined to exon 4. As the reverse primer used in our study was located between positions 1009 and 1026 within exon 3 (Green *et al.*, 1986; Ponglikitmongkol *et al.*, 1988) the presence of such a variant could result in negative findings by PCR analysis. Although exon 3 is known to encode the second zinc finger of the receptor, which is required for DNA binding, Fuqua *et al.* (1993) found that the variant with the missing exon 3 did not show any loss of wild-type transcriptional activity or DNA binding. This particular variant may therefore represent a natural alternatively spliced form of ER that is functional. Proof of this contention for variant ERs will, however, only come from functional studies such as evaluation of the response to hormonal manipulation in those patients in whom variant ERs are detected. In this regard, PCR analysis with nested primers would appear to be a suitable approach for screening for such ER variants. It should, however, be pointed out the four samples in question were all low positive by immunochemistry, with an average H score of 40. These cells may no longer have been synthesising ER protein. The one sample that was analysed as ER mRNA positive but ERICA negative could have been due to the presence of a mutation affecting the expression of the ER protein. Again further examination of the ER gene by PCR should be done to investigate this possibility. The PCR technique for examining expression of ER mRNA may therefore also provide a good screening method for detection of mutations.

The potential usefulness of PCR for the detection of TGF-β mRNA expression in breast cancer cells is confirmed by the results of the comparison between PCR and immunocytochemistry showing sensitivity and specificity of 100%. Although the significance of TGF-β expression in breast cancer has not been fully elucidated, the role of TGF-β as a negative growth regulator for hormone-sensitive breast cancer derived cell lines has been established *in vitro* (Knabbe *et al.*, 1987). Whether this model is applicable to *in vivo* human breast cancer, remains to be established and a reliable, sensitive method for detecting the transcription of the gene for this growth factor would appear to be extremely useful for such studies.

While PCR can perpetuate and exponentially amplify even minute errors in the templates it appears to be a viable technique for analysis of both ER and TGF-β. Only a small sample of material is required for analysis (as few as 1000 cells), it is a rapid technique and subjective interpretation errors and interobserver variations, which occur in immunocytochemical techniques, can be avoided.

**Acknowledgements**
This study was supported by grants from the Cancer Association of South Africa (CANSA) and the George Elkin Bequest.

### References

BARRETT-LEE P, TRAVERS M, LUQMANI Y AND COOMBES RC. (1990). Transcripts for transforming growth factors in human breast cancer: clinical correlates. *Br. J. Cancer*, **61,** 612–617.

CHOMCZYNSKI P AND SACCHI N. (1987). Single-step method of RNA isolation by acid guanidinium thiocyanate-phenol-chloroform extraction. *Anal. Biochem.*, **162,** 156–159.

CLARK GM, OSBORNE CK AND MCGUIRE WL. (1984). Correlations between estrogen receptor, progesterone receptor and patient characteristics in human breast cancer. *J. Clin. Oncol.*, **2,** 1102–1109.

COOKE T, GEORGE D, SHIELDS R, MAYNARD PV AND GRIFFITHS K. (1979). Oestrogen receptors and prognosis in early breast cancer. *Lancet*, **1,** 995–997.

DAO TL AND NEMOTO T. (1980). Steroid receptors and response to endocrine ablation in women with metastatic cancer of the breast. *Cancer*, **46,** 2779–2782.

DE SOMBRE ER, CARBONE PP, JENSEN EV, MCGUIRE WL, WELLS SA Jr, WITTLIFF JL AND LIPSETT MB. (1979). Steroid receptors in breast cancer. *N. Engl. J. Med.*, **301,** 1011–1012.

DE SOMBRE E, THORPE S, ROSE C, BLOUGH RR, ANDERSEN KW, RASMUSSEN BB AND KING WJ. (1986). Prognostic usefulness of estrogen receptor immunocytochemical assays for human breast cancer. *Cancer Res.*, **46** (suppl.), 4256S–4264S.

FUQUA SAW, ALLRED DC, ELLEDGE RM, KRIEG SL, BENEDIX MG, NAWAZ Z, O'MALLEY BW, GREENE GL AND MCGUIRE WL. (1993). The ER-positive/PgR-negative breast cancer phenotype is not associated with mutations within the DNA binding domain. *Breast Cancer Res. Treat.*, **26,** 191–202.

GLAUBER JG AND KIANG DT. (1992). The changing role of hormonal treatment in advanced breast cancer. *Semin. Oncol.*, **19,** 308–316.

GREEN S, WALTER P, KUMAR V, KRUST A, BORNERT J-M, ARGOS P AND CHAMBON P. (1986). Human oestrogen receptor cDNA: sequence, expression and homology to v-erbA. *Nature*, **320,** 134–139.

HIRAYAMA D, FUJIMORI T, SATONAKA K, NAKAMURA T, KITAZAWA S, HORIO M, MAEDA S AND NAGASAKO K. (1992). Immunohistochemical study of epidermal growth factor and transforming growth factor-β in the penetrating type of early gastic cancer. *Hum. Pathol.*, **23,** 681–685.

JONAT W, MAASS H AND STEGNER H. (1986). Immunohistochemical measurement of estrogen receptors in breast cancer tissue samples. *Cancer Res.*, **46,** 4256S–4298S.

KING WL AND GREEN GL. (1984). Monoclonal antibodies localize oestrogen receptor to the nuclei of target cells. *Nature*, **307,** 293–304.

KING WL, DE SOMBRE ER, JENSEN EV AND GREEN GL. (1985). Comparison of immunocytochemical and steroid binding assays for estrogen receptor in human breast tumours. *Cancer Res.*, **45,** 293–304.

KNABBE C, LIPPMAN ME, WAKEFIELD LM, FLANDERS KC, KASID A, DERYNCK R AND DICKSON RB. (1987). Evidence that transforming growth factor-β is a hormonally regulated negative growth factor in human breast cancer cells. *Cell*, **48,** 417–428.

KNIGHT WA, LIVINGSTON RB, GREGORY EJ AND MCGUIRE WL. (1977). Estrogen receptor as an independent prognostic factor for early recurrence in breast cancer. *Cancer Res.*, **37,** 4669–4671.

KORENMAN SG AND DUKES BA. (1970). Specific estrogen binding by the cytoplasm of breast carcinoma. *J. Clin. Endocrinol.*, **30,** 639–645.

LEVIN J, RAY G, DA FONSECA M, LANGE M, DE MOOR NG AND SAVAGE M. (1978). Oestrogen receptor in tumours of breast cancer patients. *S. Afr. Med. J.*, **53,** 447–479.

LIPPMAN ME AND ALLEGRA JC. (1980). Quantitative estrogen receptor analysis: the response to endocrine and cytotoxic chemotherapy in human breast cancer and the disease-free interval. *Cancer*, **46,** 2829–2834.

MCCARTY KS Jr, SVABO E, FLOWERS JL, COX EB, LEIGHT GS, MILLER L, KONRATH J, SOPER JT, BUDWIT DA, CREASMAN WT, SEIGLER HF AND MCCARTY KS Snr. (1986). Use of a monoclonal antiestrogen receptor antibody in the immunohistochemical evaluation of human tumours. *Cancer Res.*, **46** (suppl.), 4244S–4248S.

MCGUIRE WL, PEARSON OH AND SEGALOFF A. (1975). Predicting hormone responsiveness in human breast cancer. In *Estrogen Receptors in Human Breast Cancer*. McGuire WL, Carbone PO and Vollmer EP (eds) pp. 27–30. Raven Press: New York.

MAYNARD PV, BLAMEY RW, ELSTON CW, HAYBITTLE JL AND GRIFFITHS K. (1978). Oestrogen receptor assay in primary breast cancer and early recurrence of the disease. *Cancer Res.*, **39,** 4292–4295.

OSBORNE CK, YOCHMOWITZ MG, KNIGHT WAIII AND MCGUIRE WL. (1980). The value of estrogen and progesterone receptors in the treatment of breast cancer. *Cancer*, **46,** 2884–2888.

PARIDAENS R, SYLVESTER RJ, FERRAZZI E, LEGROS N, LECLERQ G AND HEUSON JC. (1980). Clinical significance of the quantitative assessment of estrogen receptors in advanced breast cancer. *Cancer*, **46,** 2889–2895.

PEGORARO R, KARNAN V, NIRMUL D AND JOUBERT S. (1986). Estrogen and progesterone receptors among women of different racial groups. *Cancer Res.*, **46,** 2117–2120.

PELTON RW, DICKINSON ME, MOSES HL AND HOGAN BLM. (1990). In situ hybridization analysis of TGF$\beta$3 RNA expression during mouse development: comparative studies with TGF$\beta$1 and $\beta$2. *Development*, **110**, 609-620.

PONGLIKITMONGKOL M, GREEN S AND CHAMBON P. (1988). Genomic organization of the human estrogen receptor gene. *EMBO J.*, **7**, 3385-3388.

ROBERTS AB, THOMPSON NL, HEINE U, FLANDERS C AND SPORN MB. (1988). Transforming growth factor-$\beta$: possible roles in carcinogenesis. *Br. J. Cancer*, **57**, 594-600.

ROSE C, THORPE SM, ANDERSON KW, PEDERSON BV, MOURIDSEN HT, BLICHERT-TOFT M AND RASMUSSEN BB. (1985). Beneficial effect of tamoxifen in primary breast cancer patients with high estrogen receptor values. *Lancet*, **1**, 16-18.

SEYMOUR L, MEYER K, ESSER J, MACPHAIL AP, BEHR A AND BEZWODA WR. (1990). Estimation of PR and ER by immunocytochemistry in breast cancer. *Am. J. Clin. Pathol.*, **94** (suppl 1), 35-40.

STEFANINI M, DE MARTINO C AND ZAMBONI L, (1967). Fixation of ejaculated spermatazoa for electron microscopy. *Nature*, **216**, 173-174.

THORPE SM, ROSE C, RASMASSEN BB, MOURIDSEN HT, BAYER T AND KEIDING N. (1987). Prognostic value of steroid hormone receptors and multivariate analysis of systemically untreated patients with node negative primary breast cancer. *Cancer Res.*, **47**, 6126-6133.

WITTLIFF JL. (1979). The steroid receptors of experimental mammary tumours and their relationship to those of human breast carcinoma. *Mol. Aspects Med.*, **2**, 395-437.

WITTLIFF JL. (1984). Steroid hormone receptors in breast cancer. *Cancer*, **53**, 630-643.